# X-ray Absorption Spectroscopy Characterization of a Li/S Cell

**DOI:** 10.3390/nano6010014

**Published:** 2016-01-11

**Authors:** Yifan Ye, Ayako Kawase, Min-Kyu Song, Bingmei Feng, Yi-Sheng Liu, Matthew A. Marcus, Jun Feng, Elton J. Cairns, Jinghua Guo, Junfa Zhu

**Affiliations:** 1 National Synchrotron Radiation Laboratory and Collaborative Innovation Center of Suzhou Nano Science and Technology, University of Science and Technology of China, Hefei 230029, China; yifanye@lbl.gov; 2 Advanced Light Source, Lawrence Berkeley National Laboratory, Berkeley, CA 94720, USA; bfeng@lbl.gov (B.F.); ysliu2@lbl.gov (Y.-S.L.); mamarcus@lbl.gov (M.A.M.); fjun@lbl.gov (J.F.); 3 Environmental Energy Technologies Division, Lawrence Berkeley National Laboratory, Berkeley, CA 94720, USA; akawase@lbl.gov (A.K.); ejcairns@lbl.gov (E.J.C.); 4 Department of Chemical and Biomolecular Engineering, University of California, Berkeley, CA 94720, USA; 5 School of Mechanical and Materials Engineering, Washington State University, Pullman, WA 99164, USA; mksong325@gmail.com; 6 School of Materials Science and Engineering, Harbin Institute of Technology, Harbin 150001, China; 7 Department of Chemistry and Biochemistry, University of California, Santa Cruz, CA 95064, USA

**Keywords:** lithium/sulfur cell, X-ray absorption spectroscopy, cetyltrimethylammonium bromide, synthesis, capacity decay, cycled cathode materials, insulating layer, *in-situ/in-operando*

## Abstract

The X-ray absorption spectroscopy technique has been applied to study different stages of the lithium/sulfur (Li/S) cell life cycle. We have investigated how speciation of S in Li/S cathodes changes upon the introduction of CTAB (cetyltrimethylammonium bromide, CH_3_(CH_2_)_15_N^+^(CH_3_)_3_Br^−^) and with charge/discharge cycling. The introduction of CTAB changes the synthesis reaction pathway dramatically due to the interaction of CTAB with the terminal S atoms of the polysulfide ions in the Na_2_S*_x_* solution. For the cycled Li/S cell, the loss of electrochemically active sulfur and the accumulation of a compact blocking insulating layer of unexpected sulfur reaction products on the cathode surface during the charge/discharge processes make the capacity decay. A modified coin cell and a vacuum-compatible three-electrode electro-chemical cell have been introduced for further *in-situ*/*in-operando* studies.

## 1. Introduction

Sustainable and clean energy technologies are highly desirable due to the increasing global energy consumption. The rechargeable lithium/sulfur (Li/S) cell is one option and has been regarded as a cutting-edge technology [1,2,3]. The Li/S cell has received significant attention due to the high theoretical specific capacity of 1675 mA·h/g for elemental S [3]. However, the widespread applications of the Li/S cells are limited by some issues, such as low electronic conductivity and lithium ion diffusion rate for S and sulfides; volume expansion (~76%) and the polysulfide shuttle effect [4,5]. To address these challenges, a melt-diffusion assistance method was developed to load sulfur onto conductive porous carbon materials, such as microporous carbon spheres, porous hollow carbon, porous carbon nanofibers, and graphene oxide (GO) [5,6,7,8]. Our interests are in applying GO as the conductive material, due to the advantages of high carrier mobility, high surface area and excellent chemical stability [1,2,4,6,9]. Furthermore, we developed a surface modification method by using the surface agent CTAB (cetyltrimethylammonium bromide) [1,2,6]. The CTAB-modified S-GO cell demonstrated cycling performance up to 1500 cycles with extremely low capacity decay rate of 0.039% per cycle, which meets most of the requirements for the development of high-performance cells for zero-emission vehicles [2]. However, detailed understanding of the chemical bonding and electronic structures of the cathode materials is still limited, which precludes the further improvement of the cathode materials in the Li/S cells. Furthermore, even though we obtained very good improvement of the cell performance, the capacity fading (around 60% after 1500 cycles) is still the main obstacle for further improving the cell cycle life. Therefore, it is highly desirable to acquire a deep insight on why the capacity fades on cycling by analyzing the evolution of the cathode materials after various numbers of discharge/charge cycles.

In this work, to obtain a comprehensive picture on the whole process of a Li/S cell life cycle, the X-ray absorption spectroscopy (XAS) technique was applied. The role of the CTAB was investigated by observing the synthesis process of the CTAB-S nano-composites. Samples in both liquid and solid phases can be monitored by XAS, indicating that each step at the synthesis process can be investigated using this technique, which enables us to get a much more detailed and comprehensive picture of the S speciation during the sample synthesis process. With this effort, the effect of different sample preparation procedures on the chemical structure of cathode materials can be clearly identified, which is essential for the synthesis recipe optimizations. Moreover, the structural evolution of the cathode materials based on S-GO-CTAB nanocomposites used in real Li/S cells after different charge/discharge cycles can be monitored. By employing two different detection modes (*i.e.*, total electron yield (TEY) mode and total fluorescence yield (TFY) mode during the XAS measurements, a detailed depth profile can be obtained, providing direct structural information from the near surface (which contacts with the electrolyte) and bulk of the cathode materials. This will lead us to better understand the degradation mechanism of the Li/S cell. Furthermore, by developing two *in-situ*/*in-operando* setups, we are able to *in-situ* investigate the dynamics and kinetics of the cathode materials of Li/S cells during the charge/discharge cycle. The fundamental information gained in this study will surely help the battery scientists to develop new Li/S cells with high performance.

In comparison with the other characterization tools, such as transmission X-ray microscopy, X-ray diffraction, UV-visible spectroscopy and electron paramagnetic resonance, XAS has special advantages in the studies of Li/S cell materials because it is extremely sensitive to chemical and electronic structures of materials. The lack of the chemical and electronic state information of the Li/S cathode materials has become a serious impediment for the Li/S cell cathode material optimization effort. Therefore, in this paper, we want to contribute our efforts on solving this problem by introducing XAS technique to the application on the study of a Li/S cell.

## 2. Results and Discussion

### 2.1. Introduction of X-Ray Absorption Tools

XAS is a widely used technique for investigation of geometric and electronic structures of matter [10,11,12,13,14]. It is well established that XAS allows us to determine the chemical bonding, oxidation states, band structures and local symmetries of materials [13,15]. To gain detailed information on the application of XAS, the X-ray absorption process is briefly described here.

When X-rays hit a sample, they will either be scattered or absorbed by the electrons in the sample [16]. In an X-ray absorption process, a core-hole is first created when a core-electron is excited up to an unoccupied state above the Fermi energy. This transition is governed by the dipole-selection rules that limit the available final states. The energy dependence of X-ray absorption thus depends on the distribution of unoccupied states that may be reached from the core-level via a dipole-allowed transition. The resulting core-hole can be refilled by the higher energy core-level electrons, valence electrons or previously excited electrons, with emission of photons (photon-in/photon-out process). A detector can be used to collect these photons as a TFY signal for XAS. Moreover, the dominant channel for decay of the core-hole is Auger electron emission, rather than fluorescence [17]. Hence, a low energy secondary electron cascade can be induced by the inelastic electron scattering of the Auger electron, which provides the possibility of surface sensitive detection. Therefore, we have three methods for detecting X-ray absorption signals, each with a different probe depth: Auger electron yield (AEY) is measured by detecting primary Auger electrons which have not lost energy, and is thus as surface-sensitive as X-ray photoelectron spectroscopy (XPS). TEY detection includes the secondary-electron cascade and has a probe depth of 5 nm (soft X-rays) to tens of nm (hard X-rays), limited by the depth from which electrons emerge with enough energy to overcome the work-function barrier [17]. In contrast, TFY detection probes the samples at depths of up to microns. These signals come from the different decay routes of refilling the core-hole, and therefore can be collected simultaneously. However, in most cell investigations, the sample preparation processes are mostly conducted via wet-chemistry in air while the cycled cathode materials surfaces are highly influenced by possible remaining residues and adsorbed species in the air [2,18]. Therefore, the surfaces of the materials may be complicated and highly influenced by the uncontrollable experimental environment. Hence, AEY, as well as XPS, with its high surface sensitivity, may provide misleading information and is not suitable for *in-situ*/*in-operando* studies. Thus, TEY and TFY are mostly applied to get depth profiling information [10,11,18,19]. Therefore, XAS can probe the materials chemical and electronic information ranging from several to hundreds nanometers, leading to a deep insight into the studies of the Li/S cells.

As described above, XAS can detect the speciation of S in both liquid and solid phases, and also surface and bulk information of the cathode materials. Therefore, some examples related to how XAS can help us better understanding the cathode materials of the Li/S cell will be gave in the later sections. We aimed to provide some examples demonstrating methodologies linking XAS results with practical parameters. Through this paper, we try to couple the XAS technical progress with its application in Li/S cathode materials studies.

### 2.2. Application of S K-Edge XAS to the Characterization of the Sample Synthesis Process

**Figure 1:**
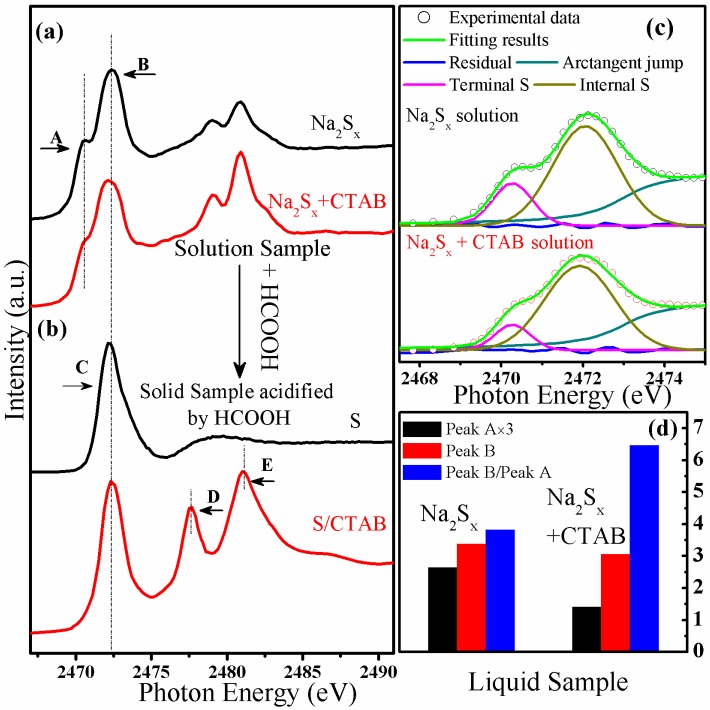
S K-edge X-ray absorption spectroscopy (XAS) data of (**a**) Na_2_S*_X_* and Na_2_S*_X_* + cetyltrimethylammonium bromide, CH_3_(CH_2_)_15_N^+^(CH_3_)_3_Br^−^ (CTAB) solutions, (**b**) the precipitates collected from the acidified solutions. The fitting of the lower energy region of the spectra of (**a**) is shown in (**c**), while the intensities of peaks A and B and the ratio of Peak B/Peak A are shown in (**d**).

**Figure 2:**
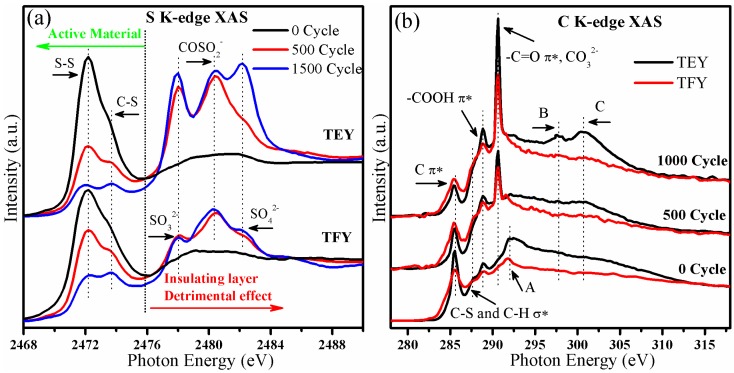
The (**a**) S K-edge XAS and (**b**) C K-edge XAS data of cathode materials cycled for 0, 500 and 1500 cycles.

Recently, Song *et al.*, and others have developed surface-modified GO (and its derivatives) based Li/S cells with excellent performance [1,2]. The surface agent CTAB is believed to play an important role during the wet-chemistry synthesis process. Therefore, we want to monitor the S speciation at each synthesis step with the introduction of CTAB. Figure 1a shows the S K-edge XAS data of Na_2_S*_x_* and Na_2_S*_x_* + CTAB solutions; while Figure 1b shows the S K-edge XAS data of the precipitates collected from the acidified solutions. The former spectra were collected with TFY mode while the later spectra were collected with TEY mode, judging from the spectra shapes, no self-absorption effect has been observed. Figure 1c shows the low-energy region of Figure 1a together with the peak fittings. And the peak intensities, and the intensity ratio are shown in Figure 1d. The peaks in the energy range of 2468–2475 eV are features of the polysulfide chains. Specifically, peaks A and B in the lower energy region arise from the differently coordinated S atoms in the polysulfide chains [20,21]. A linear polysulfide chain, S*_x_*^2−^, is represented with 1 e^−^ associated with each of the terminal S atoms, while the internal S atoms are formally uncharged. Therefore, the accumulated electronic charge at the terminal S atoms red-shifts the absorption peak with respect to the absorption peak of the internal S atoms [20]. The low photon energy features were further analyzed by fitting of the spectra with two Gaussian peaks after subtracting an arctangent edge jump. Peak A and peak B are attributed to the terminal and internal S atoms, respectively. Extracting from the fitting results shown in Figure 1c, the ratio of the intensities of Peak A:Peak B was determined to be 3.84 and 6.49 for the Na_2_S*_x_* and Na_2_S*_x_* + CTAB solutions, respectively. Accordingly, it can be estimated that the corresponding x value of Na_2_S*_x_* and Na_2_S*_x_* + CTAB are 4.7 and 6.1, respectively [22]. However, it is unexpected that the introduction of CTAB changes the length of the polysulfide chains. In addition, a comparison of the two samples shows that the internal S peak intensity slightly decreased to 90% due to the effect of the CTAB, while the terminal S peak intensity decreased up to 50%. The slight intensity deceasing of the internal S signal may be due to the concentration decreasing of S species with the introduction of CTAB. However the big intensity drop of the terminal S atoms signal can be attributed to more complicated processes. Representing CTAB in solution as CTA^+^ and Br^−^, we propose that CTA^+^ can attach to the terminal S atoms and influence the accumulated charge on the terminal S atoms, thus reducing the S^−^ (ends) signal without much affecting the signal from the internal S°. As a consequence, this interaction changed the reaction pathway and new products were obtained when precipitating the S from the solution. As shown in the Figure 1b, two new peaks, denoted as D and E, appeared with the introduction of the CTAB. These peaks vanished after heat treatment at 155 °C and a new peak at 2473.7 eV, representing the C–S bond [10,12,13], appeared (the spectra are shown in the later section, that is Figure 2a), indicating that the peaks D and E are not related to S–O bonding. Therefore, we have attributed these two peaks to the interactions of CTAB derivatives and S species. Therefore we can conclude that the introduction of CTAB can change the final products in the cathode materials synthesis process. The exact origin of these two peaks are still under discussion and further studies are needed. However, we can conclude that the CTAB was introduced by bonding with S species in the solution, and subsequently this CTAB-S component can be precipitated from the solution and further converted to C–S bond within heat treatment. The C–S bond can help to immobilize the active S during the cell cycling process [2,11,18]. Therefore, by using XAS characterization, the S speciation evolution has been clearly interpreted. And the results will benefit the understanding of the key factor of the performance enhancement for the CTAB modified Li/GO-S cell.

### 2.3. Application of S K-Edge Spectroscopy to the Characterization of Cycled Cathode Materials

S K-edge XAS has been applied to the study of the degradation mechanism of the cathode by observing the cathode materials before and after the 500th and 1500th charge and discharge cycles, respectively. The cell was stopped at the fully charged state and the cathode materials were washed with DOL-DME (DOL: 1,3-Dioxolane, DME:1,2-Dimethoxyethane) solvents three times before the measurement. TEY and TFY signals were collected simultaneously to get depth profile information. Figure 2a,b show the S K-edge XAS and C K-edge XAS of the cycled cathode materials, respectively. From the Figure 2a, two peaks can be observed in the initial cathode material: the peak at 2472.2 eV represents the transition from S 1s to the S–S π* states of S_8_ and C–S-S–C, and the peak centered at 2473.7 eV can be attributed to the transition from S 1s to the C–S σ* state [10,12,13,18]. These two peaks represent the active material that can contribute to the reaction during the charge/discharge cycle. With repeated cycling, several absorption peaks showed up in the higher photon energy region, the peaks located at 2478.0, 2480.5 and 2482.3 eV corresponding to the transition from S 1s to the σ* state of S–O in SO_3_^2−^, COSO_2_^−^ and SO_4_^2−^ [10,12,13]. These insulating species come from the reaction between Li, S and oxygen containing functional groups [10]. It can be observed from the spectra that the intensity of the S–S and C–S peaks decreased with increasing cycle number, indicating the loss of the active material. In addition, S–O species were formed and increased after cycling, at the expense of active S species. Furthermore, in comparison with the TFY signal, the ratio of peak intensities of S–S and C–S with respect to the S–O species was much lower in the TEY signal, which is surface sensitive. Therefore it can be concluded that the S–O species accumulated at the surface of the cathode materials, which is the interface of cathode materials and the electrolyte in the cell. This layer is also referred to as the SEI (solid-electrolyte interface) layer. The accumulation of this insulating SEI layer can block the diffusion of Li ions into and out of the cathode material, leading to progressive hindrance of the electrochemical reaction between Li and S, causing the cell performance to decay [10].

Meanwhile, from Figure 2b, C K-edge XAS further recorded the surface species of the cycled materials. In the initial cathode, as labeled in the figure, features at 285.5, 287.6, and 288.7 eV come from the transition from C 1s to the π* of GO and carbon black, C–S and C–H σ* and –COOH π*, respectively [10,11,23,24]. Moreover, the peak A located at 292.0 eV is attributed to the transition from C 1s to the σ* state at the *Γ* point of the graphene Brillouin zone [10,11]. On cycling, we find: (1) the damping of C π* peak at 288.5 eV and vanishing of the GO σ* peak at 292.0 eV; (2) the increase of the peak at 290.6 eV and peaks B and C at higher photon energies. The peak around 290.6 eV arises from the transition from C 1s to the –C=O π* state [25], while peaks B and C are associated with the appearance of CO_3_^2−^ [26]. Therefore, we can conclude that, with cycling, the carbon and graphene structures are highly influenced while the CO_3_^2−^ species accumulates. Moreover, similar to what we have observed on the S K-edge XAS, depth profile information can be obtained by a comparison between C K-edge TEY and TFY signals. The intensity ratio of CO_3_^2−^ peak relative to the graphene π* state is higher for the TEY signal than for TFY. This observation indicates that the accumulation of the CO_3_^2−^ species also took place at the surface of the cycled cathode material. The accumulation of this layer also probably contributed to the cell capacity decay.

Note that the intensities of the signals from these S–O species increased significantly between the initial cathode and the cathode cycled for 500 cycles. However the difference is not that significant between the cathode cycled for 500 cycles and 1500 cycles. With slight differences, significant increases in the intensity of the SO_3_^2−^, SO_4_^2−^ and CO_3_^2−^ signals were found. However, the continuous decrease of the S–S/C–S can be observed during the cycling from 500 to 1500 cycles. Therefore, we conclude that the insulating layer accumulated quickly during the first hundreds of cycles, slowing down thereafter. However, the amount of active S decreased and the CO_3_^2−^ increased at a roughly constant rate during the whole experiment. Thus, there are at least two components contributing to the overall observed capacity fade: the accumulation of insulating layer and loss of active sulfur.

The application of XAS technique provides both near-surface and bulk information at a range from several to hundreds of nanometers concerning the speciation of different elements on the cathode materials. This comprehensive picture can benefit the understanding of the Li/S cell degradation mechanism.

### 2.4. In-situ/in-Operando Experimental Setup

**Figure 3:**
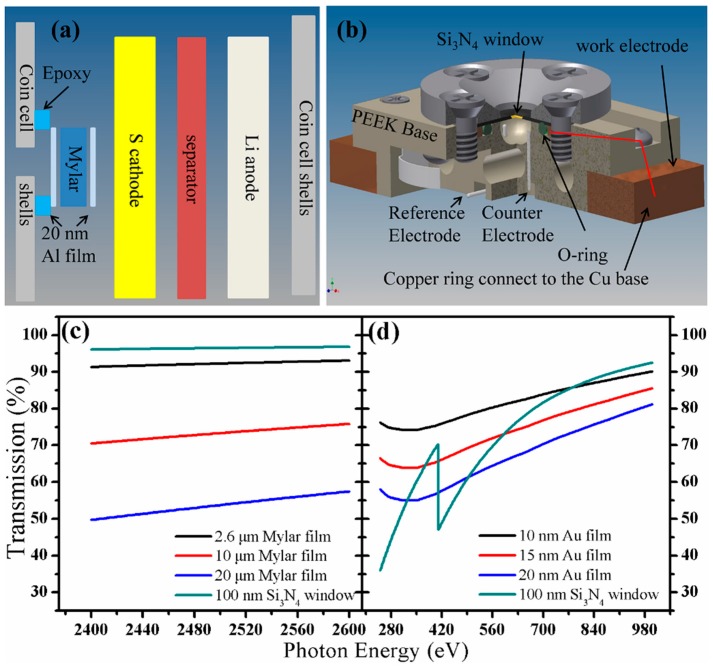
The scheme of (**a**) a modified coin cell and (**b**) a three-electrode electro-chemical cell (figure adapted from Reference [14]); and X-ray transmission at (**c**) soft X-ray energy region and (**d**) tender X-ray energy region through different materials.

The X-ray absorption process starting from the excitation of the core-level electrons makes the XAS technique highly element specific [14]. Therefore we can observe the behaviors of various elements to fulfill different experimental purposes. For example, C and O K-edge XAS can be used to investigate the interactions between the aforementioned conductive carbon based materials with S [11]. Additionally, C and O K-edge XAS can be used to investigate the degradation mechanism of the cathode side of the Li/S cells [10], one example has also been provided above. Besides these, some 3d transition metal based additives have been added to the cathode to improve the cell performances [27,28,29], and metal L-edge XAS can provide directly insight to the additive’s electronic and structural evolutions and probe the interactions among the components in the cathode materials. More importantly, S K-edge XAS can be applied to investigate S species, the major element in Li/S cells, the evolution during the sample preparation and the cell cycling process [21,30,31]. This specific information enables us get a clear and comprehensive understanding of the Li/S cell system. The C and O K-edge XAS features originating from transitions from 1s to 2p orbitals appear at energies from 280 to 320 eV and 525 to 560 eV, respectively; 3d-transition metal L-edge XAS features arising from transition from 2p to 3d show up between 350 and 1100 eV (Ca to Zn); and the S K-edge XAS peaks arising from transition from S 1s to S 3p mainly occur between 2465 and 2520 eV. The energy regions of the C and O K-edges and 3d transition metal L-edges are in the soft X-ray region, which require a vacuum system for measurement; while the energy region of the S K-edge is in the tender X-ray region which can be performed in either vacuum or ambient pressure. To get detailed information about the S speciation in the cell and the interactions of additives and the S species during the charge/discharge process, *in-situ*/*in-operando* studies are highly desirable. Several *in-situ*/*in-operando* setups have been designed, but mostly are model cells [21,30]. We have used a modified coin cell, which can provide accurate information of the nature of the electrochemical processes. A 1.0 mm × 0.5 mm rectangular hole was punched with a laser beam on the stainless steel coin cell shell. This small opening was sealed with Al coated Mylar film using epoxy. The scheme is illustrated in Figure 3a. The 2.6 μm thickness aluminized Mylar film can provide more than 90% transmission at the S K-edge, and seals the cell well. Furthermore, thicker Mylar film, such as 10–20 μm thickness Mylar film, can also be applied with good transmission of the X-ray beam, as shown in the Figure 3c. The small opening can eliminate the cell structure differences induced changes of the S speciation during the electrochemical process. The modified cell works well for tender or hard X-ray spectroscopy, but it is not vacuum-compatible. Therefore, this kind of cell cannot be used for soft X-ray absorption spectroscopy (sXAS). In previous studies, sXAS has been applied to study the LiFePO_4_ based Li-ion cell with solid electrolyte [32]. However, solid electrolytes have many limitations and are not good candidates for widespread application [33]. To address these issues, a vacuum-compatible cell was developed, illustrated in Figure 3b and described in detail in previous papers [14,34]. Basically, the cell body was built of PEEK (polyetheretherketone) material; a vacuum-sealed O-ring, a copper ring, a Si_3_N_4_ membrane window and a metal lead were used to seal the cell. A gold-coated Si_3_N_4_ membrane was attached to the copper ring and further connected to the Cu base, acting as the working electrode. Two holes at the cell bottom accommodate Al wires going through and acting as reference and counter electrodes. The holes were sealed well with epoxy. The 100 nm thickness of Si_3_N_4_ allows more than 95% transmission for X-rays at the S K-edge energy and lower but still good transmission for the energy regions of C and O K-edge and 3d transition metal L-edge. It has been well established that this design can be applied for the three-electrode electrochemical reactions. However, the applications of this cell have been highly limited into several aspects, including the catalyst reactions and some early starting of Mg-ion batteries studies. Therefore, it is highly worthwhile to check the possibility of adapting this design to the Li/S cell system by calculating the attenuation of the soft X-ray through different materials with different thicknesses. Thereafter, as described above and shown in the Figure 3d, we provided evidences that this cell design is suitable for the Li/S cell system and enable us to extend the interests to the soft X-ray regions. Moreover, this vacuum cell can also be applied to study most cathode materials used in Li-ion batteries. In conclusion, this work provided the basic perspectives on the application of the cell design on the Li/S cell system and enable us revisit and explore more details in this topic.

## 3. Experimental Section

A detailed description of the sample preparation has been reported before [2]. Basically, 0.58 g Na_2_S and 0.72 g S were dissolved in 25 mL distilled water to form a sodium polysulfide (Na_2_S*_x_*) solution after stirring with a magnetic stirrer for 2 h. 164 mg CTAB (Sigma Aldrich, St. Louis, MO, USA) was added to the Na_2_S*_x_* solution and stirred for 2 h to make a Na_2_S_x_ and CTAB mixture solution. Then, the Na_2_S*_X_* solutions with and without CTAB were slowly added to 100 mL 2 M formic acid solution (HCOOH, Sigma Aldrich, St. Louis, MO, USA), resulting in the precipitation of S species. We monitored the S-related species at all the stages involved in the synthesis of the cathode materials. The solution samples were measured using a vacuum cell sealed with 100 nm Si_3_N_4_ window that allows nearly 100% transmission at the S K-edge. A detailed description of the vacuum cell has been given above (see the Figure 3b). The CTAB modified GO-S nanocomposites were prepared using a similar method to that described above [2]. Basically, 18 mL GO dispersed in water (10 mg/mL, ACS Material, Medford, MA, USA) was diluted in 162 mL ultrapure water to make the GO suspension. The GO suspension in the presence of CTAB was added to the aforementioned Na_2_S*_x_* solution; and finally the mixed suspension was acidified with formic acid, the precipitates were the CTAB-S-GO nano-composites. After drying at 50 °C in a vacuum oven for 24 h, the nano-composites were heated in a tube furnace at 155 °C for 12 h under flowing Ar with a flow rate of 100 cc/min. The obtained nano-composites have been applied as active material, SBR/CMC were used as binders and the electrolyte is (2:1:1, *v*/*v*/*v*) PYR14TFSI/DOL/DME/0.1 M LiNO_3_ (PYR14TFSI: 1-butyl-1-methylpyrrolidinium bis(trifluoromethanesulfonyl)imide). The cell was cycled at discharge/charge rates of 1 C/0.25 C (1 C = 1675 mA/g S).

In this work, all the XAS data were taken at the Advanced Light Source, Lawrence Berkeley National Laboratory, CA, USA. For S K-edge XAS measurement, three beamlines (BLs) were used: 9.3.1, 10.3.2 and 5.3.1. BL 9.3.1 has a vacuum-chamber endstation with a beam size of 1 × 0.7 mm^2^ and energy range 2.4–5.8 keV. BL 10.3.2 has a spot size variable from 2 × 2–10 × 6 μm^2^ and energy range 2.1–17 keV, and BL 5.3.1, with a spot of around 80 × 100 μm^2^ and energy range 2.1–12 keV. For the soft X-ray, the experiment can also be performed at two vacuum based beamlines: the undulator BL 8.0.1 offers the focused high flux beam at 100 × 35 μm^2^ with energy region of 80–1200 eV, while the bend magnet BL 6.3.1.2 offers the focused beam at 50 × 500 μm^2^ with energy region of 250–2000 eV. All the beamlines allow us to measure samples using TEY and TFY modes, and BL 6.3.1.2 has the ability to record the PFY (partial fluorescence yield) signal using a Vortex (Hitachi High-Technologies Science America, Northridge, CA, USA) silicon drift detector.

## 4. Conclusions

In this work, X-ray spectroscopic techniques were introduced, with specific examples of the applications of S K-edge and C K-edge XAS to the characterization of the cathode material synthesis process and investigation of cathode material degradation mechanisms. Additionally, *in-situ*/*in-operando* setups that can fulfill different experimental purposes in the study of the Li/S cell systems were briefly introduced. With this paper, we want to establish that the XAS technique is a useful tool that can be used to get new insights on Li/S cells. Moreover, we hope the information from the XAS can offer a new strategy to explore and develop better S nanocomposite-based cathodes for advanced Li/S cells.
